# Exome array analysis of adverse reactions to fluoropyrimidine-based therapy for gastrointestinal cancer

**DOI:** 10.1371/journal.pone.0188911

**Published:** 2018-05-01

**Authors:** Matthew Traylor, Jemma L. Walker, Adele A. Corrigan, Monica A. Hernandez, Stephen J. Newhouse, Amos A. Folarin, Hamel Patel, Paul J. Ross, Jeremy D. Sanderson, James Spicer, Natalie J. Prescott, Christopher G. Mathew, Anthony M. Marinaki, Cathryn M. Lewis

**Affiliations:** 1 Department of Medical and Molecular Genetics, King’s College London, London, United Kingdom; 2 London School of Hygiene and Tropical Medicine, London, United Kingdom; 3 Purine Research Laboratory, GSTS Pathology, Guy’s and St. Thomas’ Hospital NHS Foundation Trust, St. Thomas Hospital, London, United Kingdom; 4 National Institute for Health Research (NIHR) Biomedical Research Centre for Mental Health at South London and Maudsley NHS Foundation Trust and (Institute of Psychiatry), King’s College London, London, United Kingdom; 5 Department of Gastroenterology, Guy’s and St. Thomas’ NHS Foundation Trust and King’s College London, London, United Kingdom; 6 Division of Cancer Studies, King’s College London, Guy’s Hospital, London, United Kingdom; 7 Sydney Brenner Institute for Molecular Bioscience, University of the Witwatersrand, Johannesburg, South Africa; 8 Social, Genetic and Developmental Psychiatry Centre, Institute of Psychiatry, Psychology and Neuroscience, King’s College London, London, United Kingdom; National Institute of Environmental Health Sciences, UNITED STATES

## Abstract

Fluoropyrimidines, including 5-fluororacil (5FU) and its pro-drug Capecitabine, are the common treatment for colorectal, breast, neck and head cancers—either as monotherapy or in combination therapy. Adverse reactions (ADRs) to the treatment are common and often result in treatment discontinuation or dose reduction. Factors contributing to ADRs, including genetic variation, are poorly characterized. We performed exome array analysis to identify genetic variants that contribute to adverse reactions. Our final dataset consisted of 504 European ancestry individuals undergoing fluoropyrimidine-based therapy for gastrointestinal cancer. A subset of 254 of these were treated with Capecitabine. All individuals were genotyped on the Illumina HumanExome Array. Firstly, we performed SNP and gene-level analyses of protein-altering variants on the array to identify novel associations the following ADRs, which were grouped into four phenotypes based on symptoms of diarrhea, mucositis, and neutropenia and hand-and-foot syndrome. Secondly, we performed detailed analyses of the HLA region on the same phenotypes after imputing the HLA alleles and amino acids. No protein-altering variants, or sets of protein-altering variants collapsed into genes, were associated with the main outcomes after Bonferroni correction. We found evidence that the HLA region was enriched for associations with Hand-and-Foot syndrome (p = 0.023), but no specific SNPs or HLA alleles were significant after Bonferroni correction. Larger studies will be required to characterize the genetic contribution to ADRs to 5FU. Future studies that focus on the HLA region are likely to be fruitful.

## Introduction

Fluoropyrimidines, including 5-fluororacil (5FU) and its pro-drug Capecitabine, are the common treatment for colorectal, breast, neck and head cancers—either as monotherapy or in combination therapy. Adverse reactions (ADRs) to the treatment are common and dose reduction or treatment discontinuation due to toxicity are often necessary.[1] Severe toxicity can have a rapid onset and may result in mortality in a small proportion (0.5–2%) of patients.[2, 3] Common symptoms of toxicity include diarrhea, mucositis, and neutropenia; as well as a distinctive dermatological toxic reaction known as hand-and-foot syndrome (HFS).[4] Some degree of HFS is common (50–60%) in patients treated with the fluoropyrimidine pro-drug Capecitabine, but severe reactions are more rare, occurring in 10–17% of individuals.[4]

Factors contributing to ADRs to 5FU are not well characterized. Inter-individual genetic variation is likely to contribute significantly. Variants in the dihydropyrimidine dehydrogenase (*DPYD*) gene, which encodes the primary enzyme required to metabolise fluororacil, contribute to risk of toxicity.[5, 6] However, these variants are rare, explaining only a proportion of risk, and therefore other contributing factors are likely to exist. Indeed, other associated variants have been identified using a candidate gene approach—including variants in *TYMS* and *MTHFR*.[7–9] However, the clinical relevance of these variants remains uncertain.[10] A genome-wide study of toxicity has been reported, but none of the identified associations have since been validated. [11] Genetic associations specifically with HFS have also been reported,[8, 12, 13] but large well-powered genetic studies of HFS are largely absent from the literature.

Many of the associations to date have been identified using a candidate gene approach, which has been shown to have poor reproducibility.[14] Indeed, the candidate gene approach has in general been superseded by agnostic whole-genome or whole-exome approaches, which survey the entirety of the genome or exome for associations. In this analysis, we use exome array technology to perform two analyses. First, we carry out a comprehensive assessment of the influence of protein altering variation at the SNP and gene level on ADRs to 5FU. Secondly, given the importance of the Human Leukocyte Antigen (HLA) in immune response and its prior association with drug responses,[15] we perform detailed assessment of the influence of genetic variation in the HLA on 5FU ADRs.

## Methods

### Patients and clinical data

Patient data was derived from two populations. The first was based on a series of 430 patients recruited from oncology clinics, and treated with 5FU or Capecitabine, forming part of a regional cancer network in South East London, UK; and described in detail previously. [8] Ethical approval was obtained from St Thomas’ Hospital Research Ethics Committee (07/H0802/143) and written consent was provided by all patients. For inclusion in the study, patients had to fulfil the following criteria: (1) World Health Organisation performance status <2; (2) life expectancy greater than or equal to 3 months; (3) any previous chemotherapy completed greater than or equal to 6 months ago; and (4) adequate haematological and cardiac status. Although the study was retrospective, clinical outcome data were obtained from standardised oncology outcome records completed at each clinic visit. Pre-treatment evaluation included a complete physical examination and recording of the following information: (1) baseline patient demographics (age, sex and ethnicity) and medical history; (2) diagnosis of tumour and staging (tumour, node, metastasis system); (3) current chemotherapy regimen (drug, dosing regimen) and (4) baseline blood analyses. Patients were assessed for treatment tolerance and had full blood count, renal function and liver function monitored before each chemotherapy cycle. All chemotherapy related toxicity in the first four cycles of treatment was recorded according to the National Cancer Institute Common Toxicity Criteria version 3. Patient outcome data were not disclosed to investigators undertaking the genetic analysis.

The second population consisted of 359 patients who, in addition to treatment with Capecitabine or 5-FU, had received oxaliplatin-based chemotherapy for the treatment of colorectal tumours. They were recruited from oncology outpatient clinics at Guy’s and St. Thomas’ NHS Hospital Trust, with the majority of patients (>98%) receiving treatment for colorectal carcinoma. Individuals were excluded from this analysis if this was not their first-line Capecitabine or 5FU treatment or if they had undergone previous 35 or 42 day Capecitabine cycles.

### Phenotype classification

Association was tested for two measured toxicity outcomes. Diarrhoea, mucositis and neutropenia (DMN) in the first four cycles of treatment were dichotomised as either mild to moderate (grade 0–2) or severe (grade 3–4) in all patients. In Capecitabine patients only, the primary ADR of interest was HFS. No grade 4 HFS was reported in the Capecitabine cohort, so two analyses were performed: for HFS (grade 0–1 v. grade 2–3) and for severe HFS (grade 0–2 v. grade 3). In addition we evaluated associations with Diarrhoea and Mucositis (DM), dichotomised as either mild to moderate (grade 0–2) or severe (grade 3–4) in the Capecitabine subgroup.

### Genotyping and quality control

All individuals were genotyped using the Illumina HumanExome v1.1 chip, which includes 247,870 protein-altering variants identified from whole-exome sequencing of >12,000 individuals. The array also features 2,459 HLA tags, 4,761 GWAS trait-associated SNPs, as well as ancestry-informative markers, identity-by-descent estimation markers and random synonymous SNPs to enable construction of principal components of ancestry. Comprehensive details about the exome array are available at http://genome.sph.umich.edu/wiki/Exome_Chip_Design.

Quality control (QC) of the cohort of 5FU patients was carried out on a larger dataset of 2,448 individuals, following validated procedures.[16] Manual inspection of SNP cluster plots was carried out in Genome Studio to preserve rare variants within the data. Initial call rate, gender mismatch, and visual cluster checks were also made. The SNPs were then called using zCall.[17] The above steps were applied separately to two batches (N = 1,798 and N = 650) and then merged together for further QC. SNPs were retained provided they had a call rate of 0.99 and HWE p-value >1x10^-6^. Individuals were retained provided their SNP call rate was >0.97, and any first degree relatives were removed after examining cryptic relatedness. After performing a sanity check to confirm associations of DPYD variants in the dataset as expected, we removed any individual who had any copies of four DPYD variants (c.1905+1G>A, c.2846A>T, c.1601G>A and c.1679T>G) known to be associated with ADRs.[8]

We tested for any batch effects arising from calling SNPs in two stages by (1) comparing allele frequencies; (2) testing whether loadings on principal components was correlated with batch to show that sample spreads and population structure are similar. Principal component (PCs) were calculated using *smartpca* (EIGENSTRAT) on autosomal variants pruned for linkage disequilibrium (LD) and with MAF>0.01.[18] This was first performed in combination with European, African and East Asian samples from the 1000 Genomes project. We then subset our samples on those that segregated with the European reference samples, and then repeated the principal components analysis until no individual was more than 6 standard deviations from the median on the first 5 principal components. All quality control was performed using PLINK v1.90b3.32.[19]

### HLA imputation

The Illumina HumanExome Array contains a set of 2,459 SNPs that tag variation across the HLA. We used these SNPs to impute SNPs, amino acids, and HLA alleles across the region. Imputation was performed using SNP2HLA,[20] using the Type 1 Diabetes Genetics Consortium (T1DGC) reference panel of 5,225 unrelated individuals. This resulted in imputed data relating to 728 HLA amino acids, 180 HLA alleles, and 5788 SNP genotypes.

### Statistical analysis

To assess the association of genetic variants with adverse reactions to 5FU, we performed two separate analyses; gene level and SNP level tests. Gene level tests work on the assumption that multiple variants impacting on the trait under study might reside in the same gene. These tests therefore test the aggregate impact of variants within a gene region on the trait, which can have the benefit of addition study power. [21] Conversely, SNP level tests perform a test of association of the trait with single genetic variants. We interrogated expression of potentially implicated genes using the GTEx portal (https://www.gtexportal.org/.

### Gene level tests

Gene-level tests of association were performed using the Variant Association Tools (VAT) package.[22] We used the Combined and Multivariate Collapsing (CMC) and Sequence Kernel Association (SKAT) tests to evaluate associations of variants within RefSeq genes, [21, 23] imposing default weights, filtering on MAF<0.05 and including the first two ancestry-informative principal components as covariates in all analyses. Analysis was restricted to protein-altering variants. We set the significance level to p<5x10^-6^, corresponding to Bonferroni correction for the approximate 10,000 genes in each analysis.

### SNP level tests

Associations of protein-altering SNP genotypes with each of the four ADRs were performed using PLINK v1.90b3.32, in each case including two ancestry-informative principal components to control for population structure. We assessed whether association statistics had the expected distribution by calculating genomic inflation factors and generating QQ-plots. We set the significance threshold to p<8.4x10^-7^ corresponding to Bonferroni correction for the 59,277 variants tested.

To attempt to prioritise sub-threshold associations at the SNP and gene level, we evaluated whether any of our top associations showed evidence of interaction with proteins in the DPD pathway. We used the STRING database to identify proteins with interactions with *DPYD*, *TYMS*, *DCA*, *MTHFR*, and *DPYS* with medium confidence, [24] and investigated whether any of these interacting proteins overlapped with any of our lead associations.

### HLA analysis

To interrogate the influence of HLA alleles on ADRs, we performed several analyses, as follows:

We first assessed the evidence that variation across the entire HLA region contributed to risk of each ADR. We calculated the sum of chi-squared association statistics for all genotyped SNPs across the region for each outcome, obtaining the *observed* association. We then generated 10,000 permutations of the phenotypes, and calculated *permuted* association values for each permutation, again summing chi-squared association statistics across the region. We calculated a p-value by tabulating the number of permutations at which the *permuted* sum of chi-squared association statistics equalled or exceed the *observed* association and dividing by the number of permutations.

Based on the 6696 imputed HLA alleles, amino acids and genotypes, we performed association analysis across the region for each of the four outcomes (DMN, DM, HFS, severe HFS) using PLINK v1.90b3.32, including two principal components in all analyses. We set the significance threshold at p<7.5x10^-6^, corresponding to a Bonferroni correction for all 6696 imputed elements.

## Results

After quality control procedures, our dataset consisted of 504 individuals of European ancestry (Table 1) for which information was available on 74,224 SNPs, 59,277 of which were protein-altering variants and 2,283 of which resided in the HLA region.

**Table 1:** Cohort characteristics.

	Affected			Unaffected		
	N	% male	Age (sd)	N	% male	Age (sd)
DMN	133	58.4	65.0 (11.0)	369	60.7	62.1 (12.1)
DM	50	57.1	66.0 (11.8)	199	60.7	63.3 (11.0)
HFS	36	47.2	64.4 (12.8)	218	62.4	63.8 (10.9)
Severe HFS	13	69.2	64.2 (11.4)	241	60.0	63.8 (11.2)

DM, HFS, Severe HFS considered in Capecitabine subgroup only.

### Gene level tests

We performed CMC and SKAT tests to evaluate whether genetic variation in tested genes contributed to ADRs. No genes in any of the four analyses (DMN, DM, HFS, severe HFS) reached our predetermined significant threshold (p<5x10^-6^). The ten most associated genes in each of the analyses are presented in Tables A-D in S1 File. The strongest association was for the phenotype of DMN with Unc-51 Like Kinase 4 (*ULK4*) using a SKAT test (p = 1.2x10^-5^). The strongest association with HFS phenotypes was for Phosphatidylinositol Transfer Protein, Membrane-Associated 3 (PITPNM3), which is expressed in both the transverse and sigmoid colon. [25], with severe HFS (CMC, p = 1.7x10^-4^; SKAT, p = 1.2x10^-4^).

### SNP level tests

**Fig 1:**
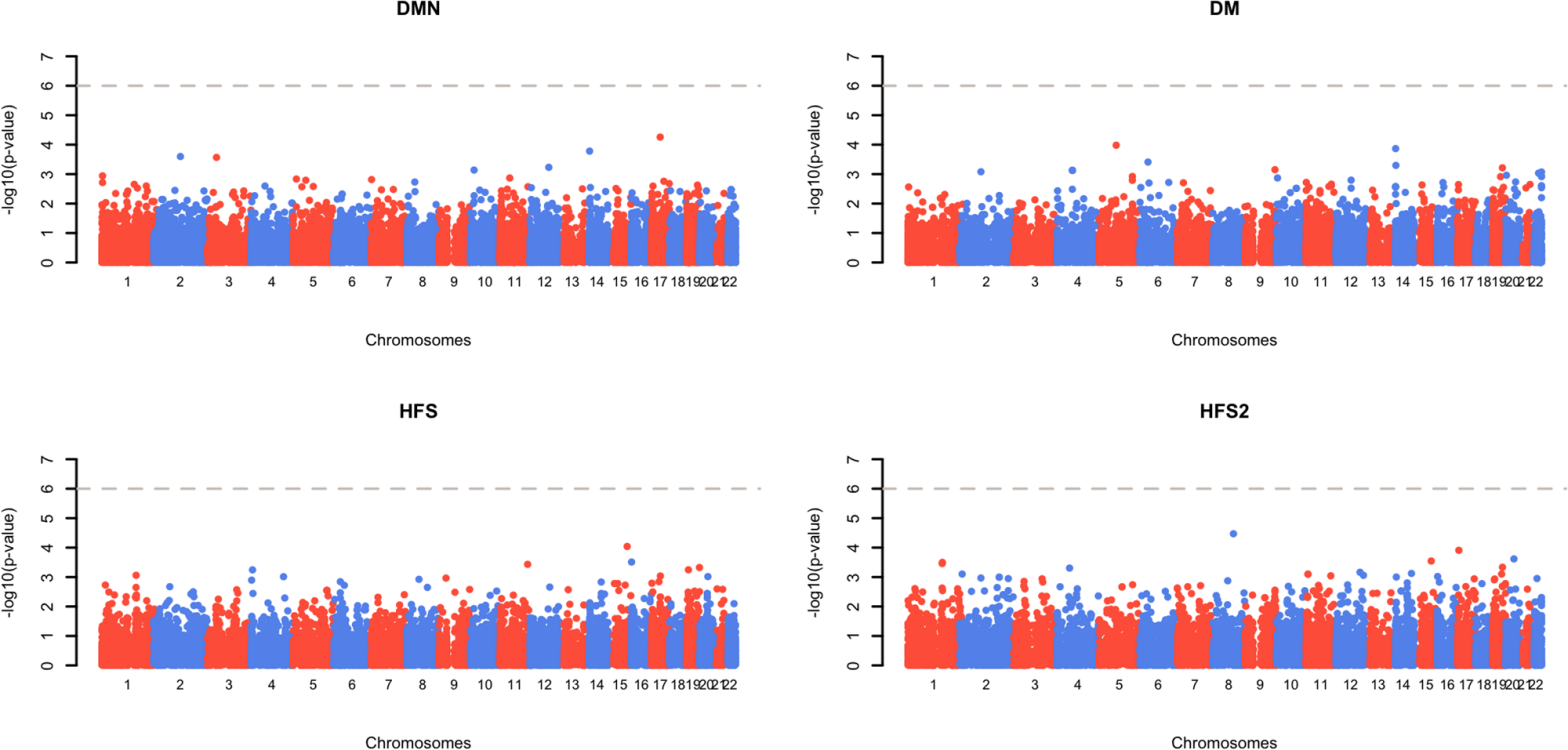
Manhattan plots of −log10(p-value) for association of genomewide protein-altering variants with DMN, DM, HFS and severe HFS by genomic position. HFS, Hand-and-Foot syndrome; DMN, Diarrhoea, mucositis and neutropenia; DM, Diarrhoea and mucositis.

Genome-wide association results for genotyped variants are presented in Fig 1. No SNP reached our significance threshold in this analysis. Genomic inflation was well controlled for all analyses (*λ* ≤ 1.01; Figure A in S1 File), suggesting no confounding due to population structure or technical artefact. The strongest associations were for exm462045, in IQ Motif Containing GTPase Activating Protein 2 (*IQGAP2)*, with DM (p = 1.0x10^-4^); and for exm709846, in Transmembrane Protein 67 (*TMEM67)*, with severe HFS (p = 3.4x10^-5^). Both are expressed in both the transverse and sigmoid colon. [25] The top associations for each of the four outcomes are presented in Tables 2 and 3.

**Table 2:** Top protein-altering SNP associations from genome-wide association analysis for ADRs.

rsID	CHR	BP	GENE	RA	RA Freq	OR	P-value
DM							
exm462045	5	75923294	IQGAP2	A	0.047	4.61	0.00010
exm1084826	14	20872881	TEP1	A	0.20	2.67	0.00014
exm543802	6	38750888	DNAH8	G	0.14	2.77	0.00039
exm1087032	14	21991626	SALL2	C	0.32	2.27	0.00050
exm1476224	19	44117052	SRRM5/ZNF428	A	0.061	3.72	0.00061
exm792722	9	136131651	ABO	A	0.082	3.64	0.00071
exm402548	4	69078113	FTLP10/TMPRSS11BNL	A	0.22	2.68	0.00075
exm402563	4	69094507	TMPRSS11BNL	A	0.22	2.68	0.00075
exm402583	4	69095197	TMPRSS11BNL	A	0.22	2.68	0.00075
exm212608	2	96861159	STARD7	G	0.16	2.61	0.00083
DMN							
exm1322587	17	39884065	HAP1	A	0.41	0.51	5.5x10^-5^
exm1084826	14	20872881	TEP1	A	0.20	1.90	0.00017
exm224289	2	121747433	GLI2	A	0.027	4.56	0.00025
exm303483	3	41759288	ULK4	G	0.030	3.97	0.00027
exm1024776	12	85432040	LRRIQ1	A	0.12	0.36	0.00059
exm813014	10	18266989	SLC39A12	G	0.34	0.56	0.00073
exm6576	1	3328659	PRDM16	A	0.13	1.93	0.0011
exm901458	11	43876698	HSD17B12	A	0.29	1.66	0.0014
exm444913	5	13737444	DNAH5	A	0.12	1.96	0.0015
exm598969	7	1595068	TMEM184A	A	0.26	1.68	0.0015

CHR, chromosome, BP, base position; RA, reference alleles; RA Freq, Frequency of reference allele; OR, odds ratio; DMN, Diarrhoea, mucositis and neutropenia; DM, Diarrhoea and mucositis.

**Table 3:** Top protein-altering SNP associations from genome-wide association analysis for ADRs.

rsID	CHR	BP	GENE	RA	RA Freq	OR	P-value
HFS							
exm1183401	15	83428192	FSD2	A	0.18	3.24	9.1x10^-5^
exm1199144	16	825255	MSLN	A	0.041	6.00	0.00031
exm967406	11	124767067	ROBO4	G	0.22	2.94	0.00037
exm1514136	19	58117083	ZNF530	G	0.15	3.17	0.00047
exm1418230	19	8176640	FBN3	A	0.22	2.70	0.00057
exm386871	4	6607046	MAN2B2	G	0.014	9.60	0.00057
exm106093	1	155172725	THBS3	G	0.11	3.17	0.00087
exm1324855	17	40722029	MLX	G	0.29	2.44	0.00092
exm1540403	20	36993333	LBP	G	0.015	15.39	0.00096
exm427090	4	146653620	C4ORF51	A	0.12	3.01	0.00097
Severe HFS							
exm709846	8	94772165	TMEM67	A	0.020	17.0	3.4x10^-5^
exm1284676	17	6386883	PITPNM3	A	0.016	51.8	0.00012
exm1540403	20	36993333	LBP	G	0.015	55.2	0.00024
exm1176020	15	74425505	ISLR2	A	0.019	14.4	0.00029
exm108413	1	156146640	SEMA4A	A	0.039	10.4	0.00032
exm106714	1	155290231	FDPS	G	0.027	10.2	0.00035
exm1476980	19	44471209	ZNF221	T	0.14	4.36	0.00046
exm400335	4	56325365	CLOCK	C	0.066	7.00	0.00050
exm1034106	12	108954862	SART3	G	0.17	5.43	0.00069
exm1121827	14	92460176	TRIP11	G	0.0030	91.5	0.00075

CHR, chromosome, BP, base position; RA, reference alleles; RA Freq, Frequency of reference allele; OR, odds ratio; HFS, Hand-and-Foot syndrome.

As *IQGAP2* has been implicated in colonic inflammation in mice, [26] we explored whether its expression was different in patients with Crohn’s Disease compared to controls, using RNA-seq data derived from the colon in 76 cases and 28 controls. *IQGAP2* showed significantly decreased expression (log(Fold Change) = -0.25; p = 2.9x10^-4^), highlighting that this gene is likely to be important in colonic inflammation.

### HLA analysis

Using a permutation-based approach, we found evidence that variation across the entire HLA region contributes to the risk of HFS (p = 0.023). Conversely, there was no evidence of association with severe HFS (p = 0.070), DMN (p = 0.20) or DM (p = 0.44).

**Fig 2:**
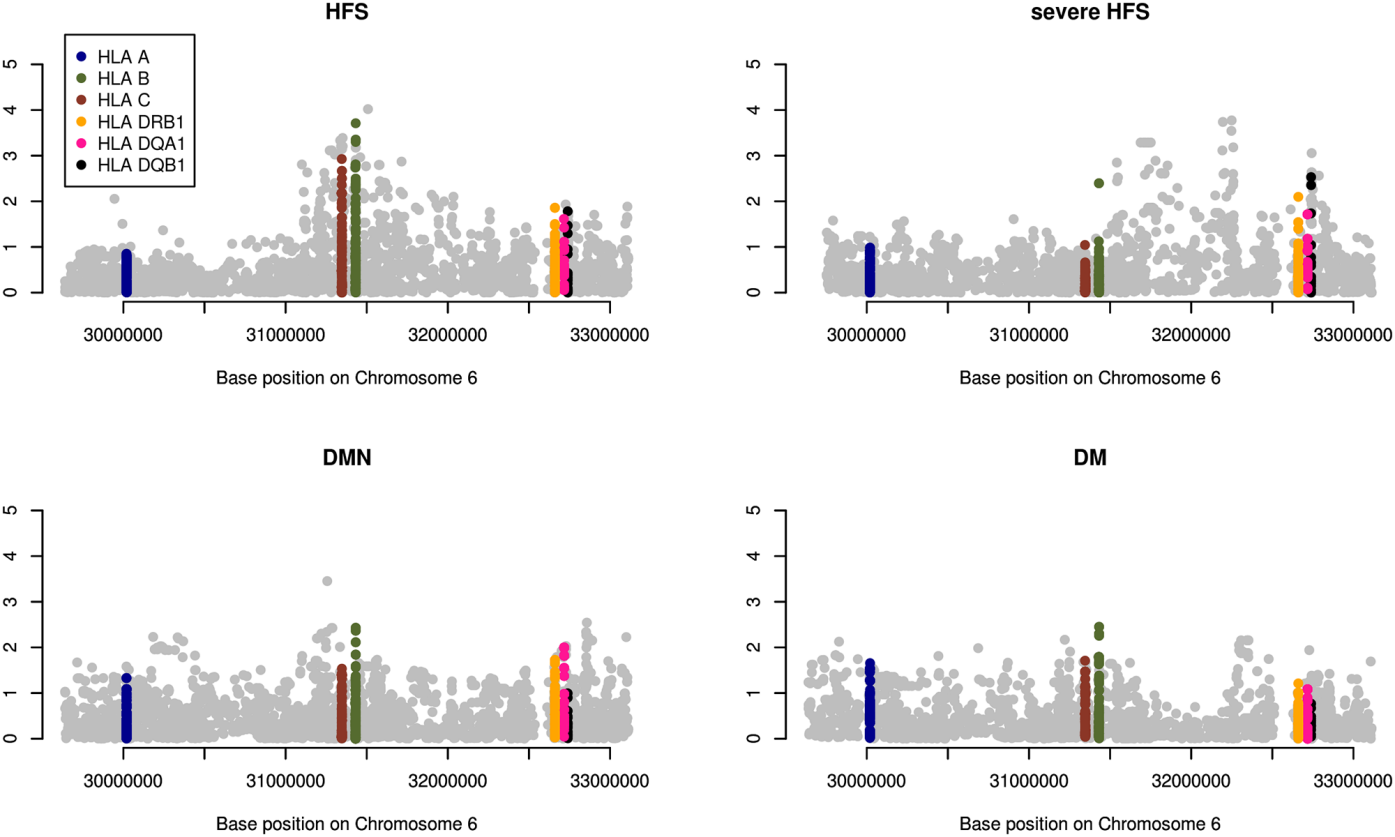
Associations of SNP variants, amino acids, and HLA alleles with HFS, severe HFS, DMN and DM across the MHC region. HFS, Hand-and-Foot syndrome; DMN, Diarrhoea, neutropenia and mucositis; DM, Diarrhoea and mucositis.

When performing association analysis of SNPs, amino acids, and HLA-alleles across the HLA region, the most significant associations were with HFS in the Class I region (Fig 2). The strongest association was with rs3093960 (OR(95% CI) = 4.5(2.1–9.5); p = 9.5x10^-5^) in the Class I region. However, no associations met the Bonferroni-correction threshold of p<7.5x10^-6^.

## Discussion

ADRs to fluoropyrimidine-based chemotherapy are a major clinical problem and often necessitate dose reduction or treatment discontinuation. Currently, dosing is based on surface area alone and does not capture inter-individual differences based on genetics or other factors. Indeed, grade 3–4 toxicity has been reported in 10–30% of individuals,[2] emphasising that current dosing strategies are unsatisfactory. Previous candidate gene studies have highlighted the importance of *DPYD* variants, [5] [6] while other studies have pointed to associations in *TYMS*, *MTHFR*, *and DCA*, which are yet to be fully validated. [8] Attempts to identify novel associations by survey of genome-wide data has so far been relateively small in scale (N = 221). [11] Here, we performed analyses using the exome array to identify genetic associations with ADRs in 430 patients undergoing fluoropyrimidine-based chemotherapy for gastrointestinal cancer.

In a comprehensive survey of protein-altering variants, we were unable to identify any novel associations with ADRs. Although none of the variants or genes studied reached the required significance level, several have functions which could plausibly implicate them in ADRs. Telomere-associated protein 1 (TEP1) was one of the strongest hits for DMN and DM. This gene plays an important role in cell survival, interacts with the BLM DNA helicase, which unwinds DNA, and may therefore have a role in DNA repair. Another interesting candidate is Semaphorin 4A (SEMA4A), which showed a suggestive association with severe HFS in our analysis. Semaphorins have been implicated in immune and inflammatory responses across many immune-mediated diseases, and SEMA4A in particular is important in stimulating immune response by activating T and B cells.[27] Such mechanisms may play a role in pathogenesis of HFS.

In addition, we performed a detailed analysis of the HLA region. Although we did not identify any specific novel associations, our permutation-based analysis across the whole HLA suggested that genetic variation across the region contributes to risk of HFS. Given the critical importance of the HLA system in immune and inflammatory response, it is very plausible that variation within the region would contribute to risk of ADRs to 5FU. Our results suggest that further detailed analysis of the region in a large, well–powered dataset are warranted.

Our study has limitations. Patients were derived from two cohorts, which we grouped together to improve power. We combined patients who had undergone treatment with 5FU or Capecitabine into a single analysis for the outcome of DMN, although Capecitabine is a prodrug for 5FU so one might expect ADRs to be consistent between the two. For HFS, severe HFS and DM, we considered only the Capecitabine subgroup. Although we aimed to survey all genome-wide protein altering variants, due to our sample size many rare variants were monomorphic in our dataset. Of the original 247,870 protein-altering variants, only 59,278 were present in the dataset. Therefore, larger samples will be required to provide a comprehensive survey of all known protein-altering variants. Other variants, in *TYMS*, *DCA* and *MTHFR*, have been reported to be associated with ADRs. These variants were not available on the exome array, meaning we were unable to investigate them in this analysis.

Our results emphasise that larger studies will be required to characterize the genetic contribution to ADRs. For affected/unaffected ratios equivalent to those in this study, 740 individuals would be required to achieve 80% power to identify variants with OR = 3 and MAF = 0.05.[28]. Additionally, more versatile statistical methods, such as machine learning, might have the potential to identify associations within datasets of this type. A collaborative effort that builds on the analysis presented herein is therefore the strategy most likely to bear fruit in the search for genetic variants contributing to ADRs to fluoropyrimidine-based therapy.

## Supporting information

S1 File 5FU_Supporting_Info.docx. (DOCX)
