# Supplementary material for: Exome array analysis of adverse reactions to fluoropyrimidine-based therapy for gastrointestinal cancer
Source: PLoS One. 2018 May 1;13(5):e0188911. doi: 10.1371/journal.pone.0188911 (PMC5929530; doi:10.1371/journal.pone.0188911)

**Supplementary Table A.** Top Associations with DMN from Gene-level analyses

| Gene | Number of variants | CMC test | | SKAT |
| --- | --- | --- | --- | --- |
|  |  | log(OR) | p-value | p-value |
| ULK4 | 9 | 0.90 | 0.0014 | 1.2x10^-5^ |
| GLI2 | 4 | 1.00 | 0.0033 | 0.00028 |
| FMO5 | 2 | 1.52 | 0.0047 | 0.00042 |
| BIVM | 2 | 1.83 | 0.0033 | 0.00062 |
| ZP3  CDH | 1 | 2.93 | 0.0070 | 0.00049 |
| CDH24 | 4 | 0.97 | 0.0018 | 0.0034 |
| TAX1BP1 | 2 | 1.84 | 0.0032 | 0.0019 |
| MYH6 | 4 | 1.50 | 0.0031 | 0.0025 |
| USP54 | 5 | 1.82 | 0.0034 | 0.0026 |
| NEK10 | 2 | 1.13 | 0.028 | 0.00035 |

**Supplementary Table B.** Top Associations with DM from Gene-level analyses

| Gene | Number of variants | CMC | | SKAT |
| --- | --- | --- | --- | --- |
|  |  | log(OR) | p-value | p-value |
| ARHGAP17 | 2 | 2.92 | 0.0013 | 6.4x10^-5^ |
| PAM | 3 | 2.55 | 0.00034 | 0.00018 |
| TAX1BP1 | 2 | 2.30 | 0.0020 | 0.00036 |
| PRKCQ | 1 | 1.94 | 0.0013 | 0.00057 |
| MOXD1 | 1 | 1.50 | 0.0016 | 0.00088 |
| DLK2 | 1 | 1.34 | 0.0051 | 0.0011 |
| CHRNE | 1 | 1.85 | 0.0020 | 0.0013 |
| SLC46A2 | 2 | 2.43 | 0.0048 | 0.0018 |
| RNF152 | 2 | 1.55 | 0.00076 | 0.0023 |
| KIAA1217 | 5 | 1.76 | 0.0016 | 0.0098 |

**Supplementary Table C.** Top Associations with HFS from Gene-level analyses

| Gene | Number of variants | CMC | | SKAT |
| --- | --- | --- | --- | --- |
|  |  | log(OR) | p-value | p-value |
| LCP | 8 | 1.45 | 0.00074 | 0.00064 |
| MOCS2 | 3 | 1.78 | 0.00038 | 0.0036 |
| MAN2B2 | 6 | 1.69 | 0.0020 | 0.00098 |
| PMPCA | 1 | 2.71 | 0.0026 | 0.0011 |
| SETD2 | 3 | 2.51 | 0.0011 | 0.0035 |
| SLC28A2 | 2 | 2.23 | 0.0048 | 0.00097 |
| PITPNM3 | 2 | 2.26 | 0.0057 | 0.0011 |
| DCAF4 | 3 | 1.49 | 0.0026 | 0.0032 |
| PSEN1 | 1 | 1.94 | 0.0036 | 0.0024 |
| C16orf3 | 1 | 2.32 | 0.0039 | 0.0024 |

**Supplementary Table D.** Top Associations with severe HFS from Gene-level analyses

| Gene | Number of variants | CMC | | SKAT |
| --- | --- | --- | --- | --- |
|  |  | log(OR) | p-value | p-value |
| PITPNM3 | 2 | 3.53 | 0.00017 | 0.00012 |
| FDPS | 1 | 2.32 | 0.00035 | 0.00035 |
| TMEM67 | 4 | 2.11 | 0.00093 | 0.00022 |
| LCN1 | 2 | 2.81 | 0.00019 | 0.0070 |
| AP1G2 | 2 | 3.46 | 0.0020 | 0.00081 |
| TMEM114 | 1 | 4.32 | 0.0015 | 0.0012 |
| AGT | 2 | 4.13 | 0.0011 | 0.0017 |
| ISLR2 | 3 | 2.19 | 0.0016 | 0.0012 |
| SEMA4A | 3 | 2.19 | 0.00027 | 0.0084 |
| LPIN1 | 3 | 2.58 | 0.0018 | 0.0013 |

**Supplementary Figure A.** QQ-plots for genome-wide SNP association statistics for ADRs


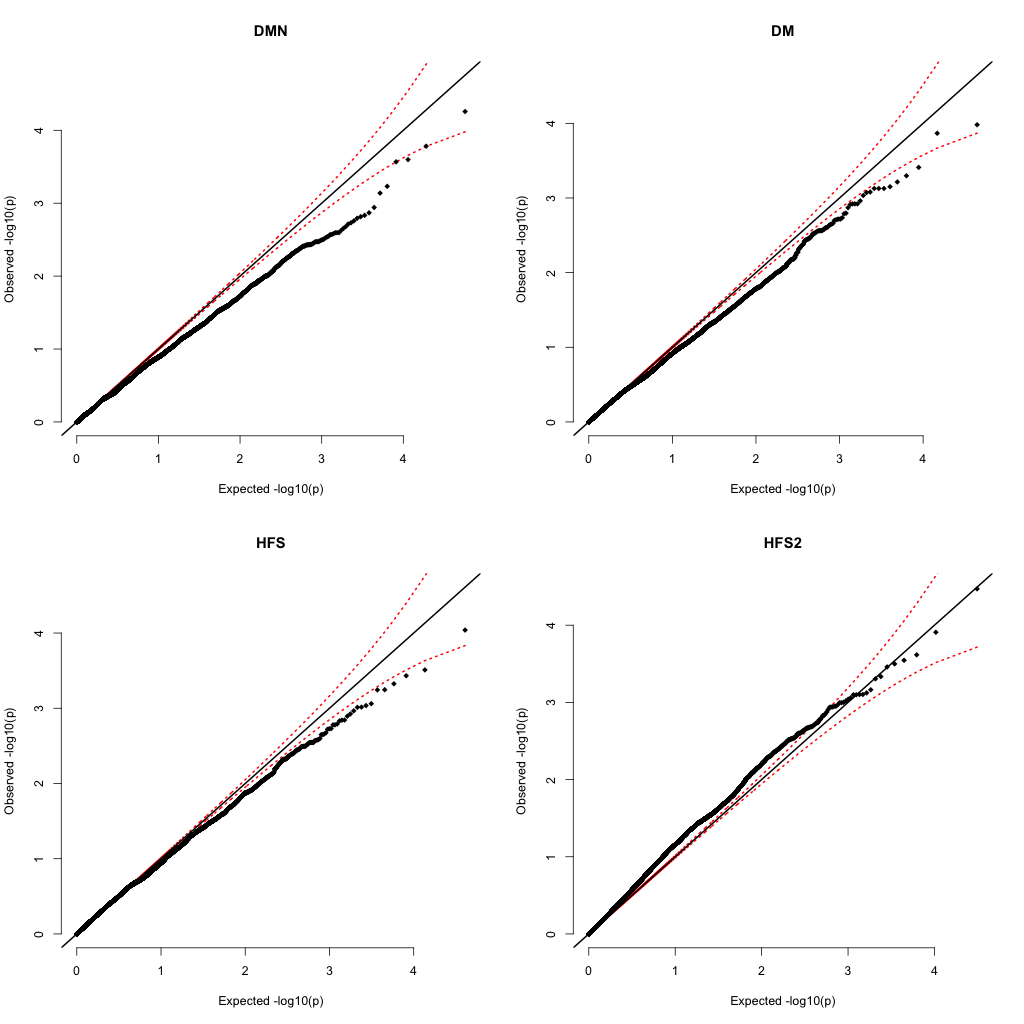

Supplement: S1 File — (DOCX) [file pone.0188911.s001.docx]
